# Supplementary material for: Myofascial urinary frequency syndrome is a novel syndrome of bothersome lower urinary tract symptoms associated with myofascial pelvic floor dysfunction
Source: Sci Rep. 2023 Oct 27;13:18412. doi: 10.1038/s41598-023-44862-5 (PMC10611808; doi:10.1038/s41598-023-44862-5)
Supplement: Supplementary file 1 — Supplementary Information. [file 41598_2023_44862_MOESM1_ESM.docx]

**Supplemental Table 1. *Multivariable Logistic Regression of Symptomatic Features Predicting Myofascial Dysfunction.***

|  |  | **vs. All**  **(With Controls)** | | | **vs. other LUTS**  **(No Controls)** | | |  | |
| --- | --- | --- | --- | --- | --- | --- | --- | --- | --- |
| **Questionnaire** | **Question** | ***RC +/- SE*** | ***p*** |  | | ***RC +/- SE*** | ***p*** |  |  |
| GUPI | 1a | 0.21 ± 1.46 | 0.888 |  | | -0.58 ± 1.61 | 0.720 |  |  |
| GUPI | 1b | -2.65 ± 1.57 | 0.092 |  | | -2.42 ± 1.71 | 0.156 |  |  |
| **GUPI** | **2b** | **2.59 ± 1.13** | **0.022** | ***** | | **2.39 ± 1.25** | **0.057** | **^.^** |  |
| *GUPI* | *2c* | *-7.66 ± 1.89* | *<0.001* | ********* | | *-7.87 ± 2.21* | *<0.001* | ***** |  |
| GUPI | 2d | 0.01 ± 1.16 | 0.996 |  | | 0.30 ± 1.48 | 0.839 |  |  |
| GUPI | 3 | 0.83 ± 0.37 | 0.025 | ***** | | 0.12 ± 0.45 | 0.796 |  |  |
| **GUPI** | **5** | **1.84 ± 0.54** | **<0.001** | ******* | | **1.49 ± 0.61** | **0.014** | ***** |  |
| OABq | 3 | 0.38 ± 0.30 | 0.201 |  | | 0.19 ± 0.33 | 0.578 |  |  |
| OABq | 4 | 0.18 ± 0.51 | 0.726 |  | | -0.02 ± 0.52 | 0.962 |  |  |
| OABq | 5 | -0.29 ± 0.35 | 0.420 |  | | -0.35 ± 0.48 | 0.422 |  |  |
| *OABq* | *8* | *-1.85 ± 0.62* | *0.002* | **** | | *-1.70 ± 0.70* | *0.016* | *** |  |
| **PFDI** | **1** | **1.04 ± 0.43** | **0.015** | ***** | | **1.19 ± 0.62** | **0.057** | **^.^** |  |
| **PFDI** | **5** | **1.75 ± 0.55** | **0.001** | ****** | | **1.54 ± 0.60** | **0.009** | ****** |  |
| PFDI | 8 | 0.18 ± 0.43 | 0.676 |  | | 0.08 ± 0.49 | 0.866 |  |  |
| PFDI | 15 | -0.18 ± 0.40 | 0.656 |  | | -0.18 ± 0.47 | 0.708 |  |  |
| PFDI | 16 | -0.42 ± 0.48 | 0.387 |  | | -0.92 ± 0.61 | 0.134 |  |  |
| PFDI | 17 | -0.51 ± 0.38 | 0.182 |  | | -0.31 ± 0.43 | 0.470 |  |  |
| PFDI | 18 | 0.33 ± 0.57 | 0.568 |  | | 0.16 ± 0.58 | 0.783 |  |  |
| PFDI | 19 | -0.22 ± 0.40 | 0.586 |  | | -0.41 ± 0.46 | 0.372 |  |  |


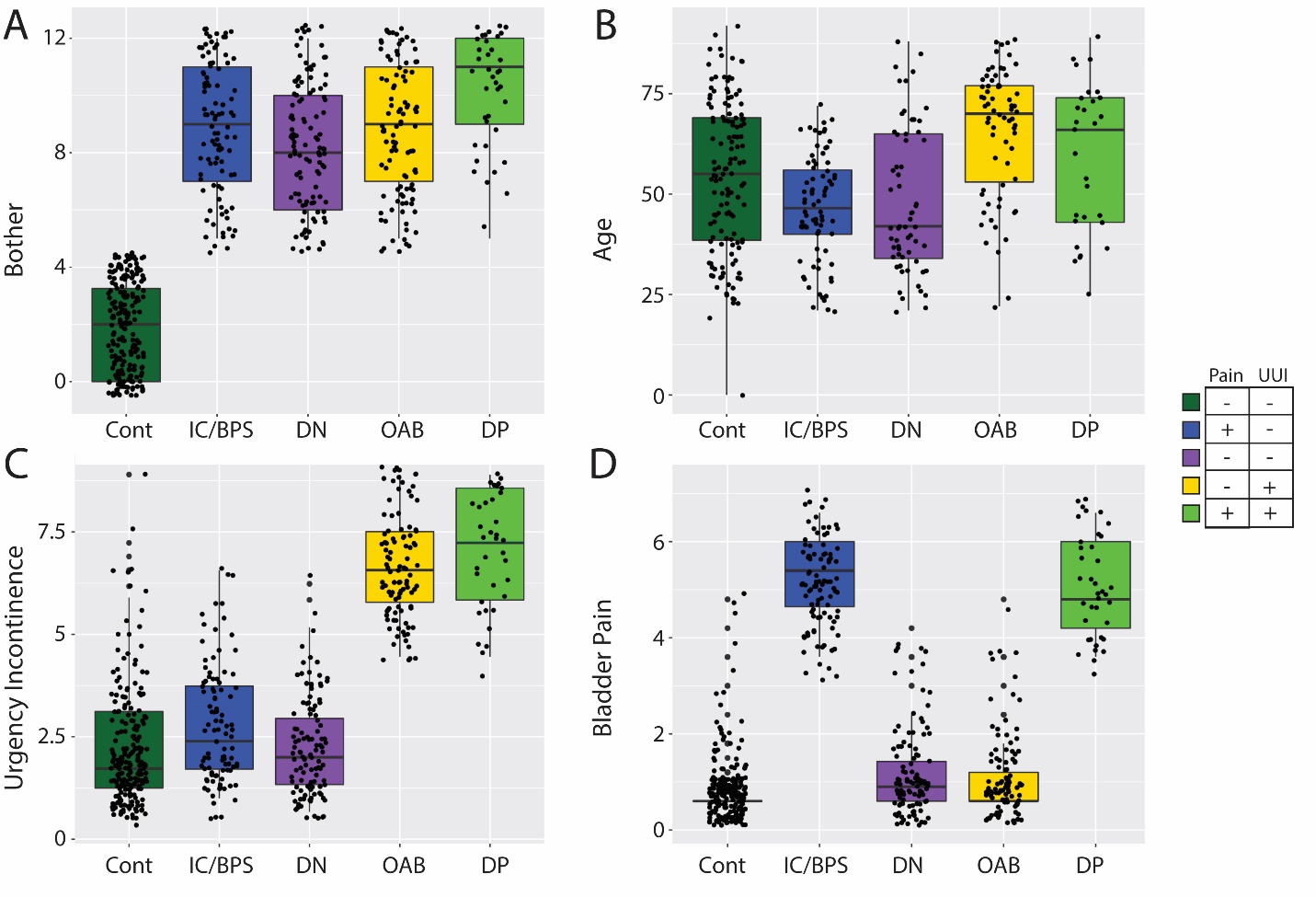


**Supplemental Figure 1.** ***Symptomatic Features of the Exploration cohort.*** Scores for overall bother (A), ages (B), urgency incontinence (C; UICI), and bladder pain (D; BPCI) were compared for the highly bothered subjects in each of the four quadrants in comparison to control subjects. Cont: controls; DN: UUI- BP-; OAB: overactive bladder (UUI+); DP: UUI+, BP+; IC/BPS: interstitial cystitis/bladder pain syndrome (BP+). UUI: Urgency Urinary Incontinence. BP Bladder Pain.
